# Markers of physiological stress during exercise under conditions of normoxia, normobaric hypoxia, hypobaric hypoxia, and genuine high altitude

**DOI:** 10.1007/s00421-017-3573-5

**Published:** 2017-03-15

**Authors:** David Richard Woods, John Paul O’Hara, Christopher John Boos, Peter David Hodkinson, Costas Tsakirides, Neil Edward Hill, Darren Jose, Amanda Hawkins, Kelly Phillipson, Antonia Hazlerigg, Nicola Arjomandkhah, Liam Gallagher, David Holdsworth, Mark Cooke, Nicholas Donald Charles Green, Adrian Mellor

**Affiliations:** 1 Royal Centre for Defence Medicine, Birmingham, UK; 2 Research Institute, for Sport, Physical Activity and Leisure, Leeds Beckett University, Leeds, UK; 3 University of Newcastle, Newcastle upon Tyne, UK; 4 Northumbria and Newcastle NHS Trusts, Wansbeck General and Royal Victoria Infirmary, Newcastle, UK; 5 Department of Cardiology, Poole Hospital NHS Foundation Trust, Poole, UK; 6 Department of Postgraduate Medical Education, Bournemouth University, Poole, UK; 7 RAF Centre of Aviation Medicine, RAF Henlow, Bedfordshire, SG16 6DN UK; 8 Imperial College London, London, UK; 9 Charing Cross Hospital, London, UK; 10 Pathology, Poole Hospital NHS Foundation Trust, Poole, UK; 11 Department of Biochemistry, Freeman Hospital, Freeman Road, Newcastle upon Tyne, UK; 12 School of Social and Health Sciences, Leeds Trinity University, Leeds, UK; 13 James Cook University Hospital, Middlesbrough, TS4 3BW UK

**Keywords:** High altitude, Hypobaric hypoxia, Normobaric hypoxia, NT-proBNP, Adrenal, Cardiac, Exercise

## Abstract

**Purpose:**

To investigate whether there is a differential response at rest and following exercise to conditions of genuine high altitude (GHA), normobaric hypoxia (NH), hypobaric hypoxia (HH), and normobaric normoxia (NN).

**Method:**

Markers of sympathoadrenal and adrenocortical function [plasma normetanephrine (PNORMET), metanephrine (PMET), cortisol], myocardial injury [highly sensitive cardiac troponin T (hscTnT)], and function [N-terminal brain natriuretic peptide (NT-proBNP)] were evaluated at rest and with exercise under NN, at 3375 m in the Alps (GHA) and at equivalent simulated altitude under NH and HH. Participants cycled for 2 h [15-min warm-up, 105 min at 55% Wmax (maximal workload)] with venous blood samples taken prior (T0), immediately following (T120) and 2-h post-exercise (T240).

**Results:**

Exercise in the three hypoxic environments produced a similar pattern of response with the only difference between environments being in relation to PNORMET. Exercise in NN only induced a rise in PNORMET and PMET.

**Conclusion:**

Biochemical markers that reflect sympathoadrenal, adrenocortical, and myocardial responses to physiological stress demonstrate significant differences in the response to exercise under conditions of normoxia versus hypoxia, while NH and HH appear to induce broadly similar responses to GHA and may, therefore, be reasonable surrogates.

## Introduction

Performing field research at high altitude (HA) is challenging and is often compounded by an austere environment and confounders such as variable environmental temperature, sleep, and exercise intensity. To mitigate these factors, the hypoxia of HA may be simulated. This may be done by reducing barometric pressure while maintaining the inspired fraction of oxygen at 20.9% (hypobaric hypoxia, HH) or by maintaining barometric pressure at 760 mmHg and reducing the inspired fraction of oxygen (normobaric hypoxia, NH). The modality employed will, pragmatically, often be determined by institutional history and availability.

Whether NH and HH are interchangeable means of simulating HA is not clearly understood, and yet, the literature is populated by studies examining the response to NH and HH with little consideration for whether there is any differential effect (Lundby et al. 2005; Woods et al. 2011b; Wille et al. 2012; Li et al. 2016). However, as illness at HA is relatively common and the pathophysiology of acute mountain sickness poorly understood (Schmerbach and Patzak 2013), there remains a need for both field and simulation studies. In 2012 (Millet et al. 2012a, b; Mounier and Brugniaux 2012), it was suggested that HH represented a more severe environmental condition that induces different physiological responses to NH (Millet et al. 2012a, b). This perspective was vigorously contested (Mounier and Brugniaux 2012) and stimulated significant debate within the field (Girard et al. 2012; Millet et al. 2012b). This debate highlighted a significant knowledge gap and the limited evidence-base on which any firm conclusions could be based. Among the very few studies comparing NH and HH, this journal has reported that HH may elicit a lower arterial carbon dioxide, lower oxygen saturation, and greater alkalosis than NH during an acute (30 and 40 min) exposure to an equivalent of 4500 m (Savourey et al. 2003, 2007), an effect thought to be due to increased dead space ventilation. However, there remains little direct comparison between the effects of NH, HH, and a “real-world” equivalent altitude, particularly in relation to the physiological stress induced. This was emphasized again when a recent review of studies utilizing NH and HH reiterated the limitations of the existing literature (Coppel et al. 2015).

We, therefore, chose an array of biochemical markers that would reflect a breadth of physiological responses to the environment at rest and with exercise allowing comparison between those environments. We, and others, have previously shown that highly sensitive cardiac troponin T (hscTnT), N-terminal pro-brain natriuretic peptide (NT-proBNP), and cortisol are influenced by hypoxia and exercise (Rostrup 1998; Banfi et al. 2010; Richalet et al. 2010; Woods et al. 2011a, 2012a, b; Mellor et al. 2014; Heinonen et al. 2014; Pagé et al. 2015; Li et al. 2016). An increased sympathoadrenal response has been reported with both acute and more prolonged HA exposure (Mazzeo et al. 1995) that is further stimulated by exercise (Mazzeo et al. 1991, 1995, 2000; Rostrup 1998). While assay of the traditional catecholamines dominates the literature, they have a very short half-life of 1–2 min (Peaston and Weinkove 2004) and are highly unstable and so labile they alter in response to a change in posture (Raber et al. 2000). Plasma normetanephrine (PNORMET, also known as normetadrenaline) and plasma metanephrine (PMET, also known as metadrenaline) are the more stable *O*-methylated extraneuronal metabolites of noradrenaline and adrenaline respectively. PNORMET and PMET, while being non-functional in themselves, both correlate with their respective catecholamines (Roden et al. 2001) and have the added advantage that they are not significantly influenced by the time of day, the phase of the menstrual cycle, or the act of venepuncture, and are stable, once separated, at 4 °C for 72 h (Deutschbein et al. 2010). Although they do not reflect global sympathoadrenal activity, they are sufficiently sensitive to increase in response to brief (2 min) high intensity exercise in normoxia (Bracken and Brooks 2010) and in view of their advantages for field work were chosen for assay rather than catecholamines.

These markers were then studied to investigate whether there was a differential response following exercise under conditions of normobaric normoxia (NN), NH, HH, and genuine high altitude (GHA).

## Materials and methods

### Ethical information

The study was approved by the Ministry of Defence Research Ethics Committee and was conducted according to the standards of the declaration of Helsinki. All participants gave written informed consent.

### Participants

Participants were 14 healthy British military servicemen and women aged 22–35 years. Participant health status was confirmed by clinical history, examination, ECG, and transthoracic echocardiography.

### Study protocol

All participants underwent a standard incremental cycle test to volitional exhaustion at sea level (absolute altitude ~113 m) under NN conditions to determine maximal oxygen uptake and maximal workload (Wmax [watts]) on a bicycle affixed to a bicycle trainer (Compu Trainer Pro Lab, Racer Mate, USA). The bicycle trainer was calibrated following the manufacturer’s instructions. At least 7 days later, this was repeated under NH {FiO_2_ considering water vapour partial pressure and daily fluctuations of barometric pressure equivalent to 3375 m (PiO_2_ ~95 mmHg)} in an environmental chamber (TISS, Alton, UK and Sporting Edge, Sherfield on Loddon, UK) at Leeds Beckett University. This established and ensured that equivalent workloads were used for the hypoxic experimental trials.

Participants were then assessed under four different environmental conditions. They were first assessed at GHA in order that conditions experienced there could then be replicated under NH and HH. GHA was at 3375 m (barometric pressure 506.4 ± 1.4 mmHg) at the Torino Hut in Northern Italy. Access to the hut required rapid ascent by cable car to 3375 m. Participants slept in the valley floor the night before, only ascending to 3375 m on the morning of their testing. Participants then remained at the hut for 24 h, descending the following day after testing. At least 7 weeks later, participants underwent the same protocol under conditions of NN. Eight weeks after being at GHA, the participants underwent the same protocol under conditions of NH in an environmental chamber. Finally, at least 3 weeks following NH exposure, participants completed the protocol under conditions of HH at the Centre of Aviation Medicine, RAF Henlow, UK. This sequence ensured that the PiO_2_ experienced breathing ambient air during GHA (PiO_2_ = 96.3 ± 0.4 mmHg) could be replicated for each individual during subsequent NH and HH exposures.

During each exposure participants had 30 min of altitude acclimatization. This was followed by a standardized warm-up which included the calibration of the bicycle trainer (Compu Trainer Pro Lab, Racer Mate, USA) to the manufacturer’s instructions. Participants then completed 120 min of cycling: 5 min at 40% Wmax, 5 min at 45% Wmax, 5 min at 50% Wmax, and 105 min at 55% Wmax. The protocol was always performed following an overnight 12-h fast and energy intake was kept uniform by the ingestion of a fixed carbohydrate solution (glucose–fructose) throughout. Participants repeated their trials at the same time of day in each environmental condition to avoid any influence of circadian variance. The temperature range across the exposures was 18–23 °C.

### Blood samples

Venous blood samples were drawn from an indwelling venous catheter in the arm following the acclimatization process, immediately prior to cycling (T0), immediately at the end of the 120 min cycling (T120), and then 2-h post-exercise (T240). At GHA, a further sample was taken 22 h later. Samples were drawn into EDTA (plasma metanephrine and normetanephrine, NT-proBNP) and SST (hscTnT, cortisol) tubes. Samples were then centrifuged and plasma and serum separated before being stored briefly at −20 °C and then at −80 °C before analysis.

### Biochemical assays

Highly sensitive cardiac troponin T (hscTnT) and N-terminal pro-brain natriuretic peptide (NT-proBNP) were assayed by the biochemistry department at Poole Hospital, Poole, UK. Cortisol and plasma metanephrines were assayed in the Department of Blood Sciences, Royal Victoria Infirmary, Newcastle upon Tyne.

NT-proBNP was measured using the Roche NT-proBNP assay (Roche Diagnostics, Mannheim, Germany) with a range from 5 to 35,000 pg/ml and a coefficient of variation (CV) at a mean NT-proBNP of 474 pg/ml of 5.8%.

HscTNT was measured using an electro-chemiluminescence immunoassay (ECLIA) on a Cobas Analyser (Roche Diagnostics). The upper reference limit (99th percentile) for this assay in healthy volunteers is 14 ng/l (pg/ml). This assay has a range from 3 to 10,000 ng/l. The CV at a mean hscTnT level of 13.5 ng/l is 5.2, 8.5% at 5.3 ng/l, and 1.8% at 28.5 ng/l.

Cortisol was measured using the Roche Elecsys Cortisol I assay which is a competitive chemiluminescence immunoassay (CLIA) that is fully automated and run on the Roche modular E unit (Roche Diagnostics, Burgess Hill, UK). The analytical range of the assay is 0.5–1750 nmol/l with an intra-assay CV of 9.3–11.7%.

Plasma metanephrine assay was performed by LC–MSMS. Following off-line solid phase using weak ion exchange extraction in a 96-well plate format, plasma metanephrines are separated using rapid, hydrophilic interaction liquid chromatography. Mass spectrometry detection is performed in multiple-reaction monitoring mode using a tandem quadrupole mass spectrometer (Waters UK, Hertfordshire, UK) with positive electrospray ionization. As well as the sample, a quality control (QC) and internal standard are used. The lower limit of quantification is between 40 and 50 pmol/l with a CV of 13–16%.

### Physiological measurements

Peripheral oxygen saturation (SpO_2_) in the finger was recorded using a Nellcor N-20P pulse oximeter (Nellcor Puritan Bennett, Coventry, UK) and heart rate was measured form a polar heart rate monitor. Measurements were taken at rest, 15 min into the acclimatization process, and then 15- and 120-min post-exercise.

### Statistical methods

Data were analysed using GraphPad Prism version 6.00 for Windows, GraphPad Software, La Jolla California USA, http://www.graphpad.com. The Shapiro–Wilk normality test and inspection of the data were undertaken to assess normality of all continuous data. All data are presented as mean ± standard deviations. Time-dependent changes within each group were assessed using repeated-measures ANOVA with Bonferroni post-test for parametric data and Friedman Test with Dunn Post-test for non-parametric data. To compare the hypoxic environments, a two-way ANOVA was performed using data only from the three hypoxic environments. A two tailed *p* value <0.05 was considered statistically significant for all comparisons.

## Results

Of the original 14 participants (age: 25.9 ± 3.8 years, height: 174 ± 10 cm, weight: 71.5 ± 10 kg, BMI: 23.4 ± 1.8 kg/m^2^, 8 M, 6 F) who completed the protocol at GHA, 13 completed the protocol under NN and NH conditions. Eight completed the protocol under HH conditions. There were no significant differences in baseline demographics between the groups. The HH exposure took place during the autumn/early winter and several participants were unable to take part due to upper respiratory tract infections and an inability to clear their ears which are exclusions to entering the hypobaric chamber.

Heart rate (beats per minute) increased from T0 to T120 in all environments with no difference between environments: NN from 67 ± 11 to 163 ± 12; GHA from 74 ± 13 to 164 ± 11; NH from 73 ± 11 to 161 ± 9; and HH from 71 ± 9 to 142 ± 24. SpO_2_ (%) dropped significantly (*p* < 0.01) with exercise: NN from 99 ± 1 to 96 ± 1; GHA from 89 ± 3 to 82 ± 4; NH from 93 ± 2 to 87 ± 4; and HH from 90 ± 2 to 83 ± 5, before returning to baseline at T240.

Data for PNORMET, PMET, cortisol, hscTnT, and NT-proBNP under conditions of NN, GHA, NH, and HH are shown in Table 1. The only significant difference in resting values (T0) was for PNORMET between NN and GHA (*p* < 0.01). NN differed from the three hypoxic environments in that only PNORMET and PMET showed a significant rise with exercise. Only at GHA was a sample taken 22-h post-exercise. PNORMET, PMET, hscTnT, and cortisol had returned to baseline 22-h post-exercise, but NT-proBNP was significantly elevated compared to baseline (36.75 ± 39 vs. 48.2 ± 61, pg/ml, *p* < 0.01).

**Table 1:** Data (mean ± SD) for PNORMET (plasma normetanephrine), PMET (plasma metanephrine), cortisol, hscTnT (highly sensitive cardiac troponin T), and NT-proBNP (N-terminal pro-brain natriuretic peptide) under conditions of NN (normobaric normoxia), GHA (genuine high altitude), NH (normobaric hypoxia), and HH (hypobaric hypoxia)

	NN (*n* = 13)			GHA (*n* = 14)				NH (*n* = 13)			HH (*n* = 8)		
	T0	T120	T240	T0	T120	T240	22 h post	T0	T120	T240	T0	T120	T240
PNORMET (pmol/l)	266 ± 193	878 ± 279*	410 ± 279	525 ± 155	1635 ± 645*	766 ± 350	482 ± 193	369 ± 101	1111 ± 442*	603 ± 279	408 ± 95	988 ± 463*	567 ± 233
PMET (pmol/l)	190 ± 75	269 ± 78*	139 ± 41	222 ± 57	359 ± 114*	173 ± 45	201 ± 48	233 ± 70	320 ± 83*	219 ± 54	244 ± 69	325 ± 75	234 ± 62
Cortisol (nmol/l)	450 ± 196	592 ± 191	327 ± 139	520 ± 276	786 ± 234*	456 ± 218	404 ± 148	519 ± 164	681 ± 189*	374 ± 153	420 ± 196	735 ± 293*	495 ± 282
hscTnT (ng/l)	3.4 ± 1.2	3.4 ± 1.3	4.3 ± 1.7	3.3 ± 1.3	4.8 ± 3.4	15.4 ± 13.6**	4.8 ± 6	3.1 ± 0.3	5.4 ± 4.2	17.3 ± 17.2**	3 ± 0	4 ± 2.6	11.5 ± 12.5**
NT-proBNP (pg/ml)	29 ± 28	30 ± 18	30 ± 29	37 ± 39	48 ± 49*	42 ± 44**	48.2 ± 61***	37 ± 38	49 ± 48*	47 ± 44**	25 ± 30	31 ± 37	32 ± 35

*Significant (*p* < 0.05) rise by T120 over baseline

**Significant (*p* < 0.05) rise by T240 over baseline

***Significant (*p* < 0.05) rise by 22 h post-exercise over baseline

Comparing the three hypoxic environments by two-way ANOVA (Table 2) revealed a significant (*p* < 0.0001) main effect of time on PNORMET, PMET, hscTnT, and cortisol but not NT-proBNP. The only significant effect for mode of hypoxia was with PNORMET (*p* = 0.0017) with no interaction between time and mode of hypoxia.

**Table 2:** Results of a two-way repeated-measures ANOVA comparing the main effects of time and mode of hypoxia across the three different hypoxic environments

	Mode of hypoxia		Time		Interaction	
	*F*	*p* value	*F*	*p* value	*F*	*p* value
PNORMET	6.88	0.0017	39.73	<0.0001	1.65	0.17
PMET	0.36	0.7	24.59	<0.0001	1.24	0.3
Cortisol	0.65	0.53	16.44	<0.0001	0.76	0.56
hscTnT	0.65	0.53	16.5	<0.0001	0.32	0.86
NT-proBNP	1.26	0.29	0.47	0.63	0.028	0.998

## Discussion

To our knowledge, this is the first study to compare the effect of NN, NH, HH, and GHA in the same participants at rest and following exercise on a range of biochemical markers that might broadly reflect physiological stress. As might be expected, the hypoxic conditions induced a different response to NN with significant rises in all studied biomarkers, whereas only PNORMET and PMET rose with exercise in NN.

Plasma metanephrines are stable markers of catecholamine secretion and have previously been reported to reflect physiological stress and the sympathoadrenal response to cycling exercise (Raber et al. 2003). The fact that PNORMET increased under all experimental conditions with exercise suggests that the protocol was sufficiently provocative to induce the required physiological stress. When comparing hypoxic environments, the only significantly different response was in that of PNORMET, suggesting that there may be subtle differences between environments in the sympathoadrenal response.

While PNORMET and PMET reflect the sympathoadrenal response, cortisol is an adrenal marker of physiological stress. We found no difference in resting cortisol between the altitude environments and NN. This is consistent with our previous data showing no change in resting cortisol at moderate altitude (Woods et al. 2012b) and others at both 3000 m (Bouissou et al. 1988) and 4300 m (Maher et al. 1975), although it is in contrast with others that have shown an increase with rapid ascent to higher altitudes at 4760 m (Sutton et al. 1977) and 6000 m (Okazaki et al. 1984). We found no rise in cortisol with exercise in NN, and while some report a rise in cortisol with exercise at SL (Bouissou et al. 1988; Wahl et al. 2014), this is not a universal finding (Hough et al. 2011; Ros et al. 2011). We did find cortisol increased significantly immediately following exercise under all hypoxic conditions.

The fact hscTnT rose 2 h following exercise under all hypoxic conditions but not in NN indicates that the hypoxic protocols were also provocative enough to induce significant myocardial TnT release. We have previously reported a rise in cTnT with exercise at HA (Boos et al. 2014; Mellor et al. 2014), as have others (Li et al. 2016). cTnT is a highly specific marker of myocardial cell damage which is released in a biphasic fashion following myocardial ischaemia with an initial small rise about 2 h after the event, thought to reflect release from the cytosolic pool, and a subsequent larger rise 12–24 h later, thought to reflect release of bound cTnT and myocardial damage (Wu et al. 1998). The fact that hscTnT had returned to baseline 22 h after exercise at GHA suggests that no myocardial necrosis had occurred and that the rise at T240 most likely reflects cytosolic release. This would be consistent with the data from our previous studies at HA that showed that while cTnT elevation was associated with higher pulmonary artery systolic pressure, there was no overt deleterious cardiac function detected by cardiac echo (Boos et al. 2014; Mellor et al. 2014).

NT-proBNP rose immediately following exercise and stayed significantly elevated 2 h later under conditions of GHA and NH, but it did not change significantly in NN. While a rise in NT-proBNP with exercise at SL has been reported, this has generally been following prolonged endurance exercise (such as running an ultramarathon) (Scharhag et al. 2005; Hew-Butler et al. 2008) rather than after moderate or short duration exercise (Nishikimi et al. 1997; Hew-Butler et al. 2008; Woods et al. 2011a) such as employed here.

We have previously reported a rise in BNP following exercise at GHA (Woods et al. 2011a, 2012a; Mellor et al. 2014). Myocardial hypoxia, intracellular acidosis, and cardiomyocyte stretch all contribute to increase NT-proBNP (Nishikimi et al. 2011). When NT-proBNP is elevated due to myocardial ischaemia and necrosis, the peak (similar to hscTnT) occurs 12–24 h later (Nishikimi et al. 2011). Interestingly, while hscTnT fell back to baseline at 22-h post-exercise, NT-proBNP remained elevated, though did not rise further. Pressure overload in the right atrium and ventricle stimulates NT-proBNP regardless of aetiology (Nishikimi et al. 2011) and we have also shown an association between NT-proBNP and high pulmonary artery systolic pressure (PASP) at HA (Banfi et al. 2010; Woods et al. 2013). The lack of a rise under conditions of HH warrants further comment. We have previously examined the BNP response to acute HH on two occasions: first, at a simulated altitude of 5334 m for 40 min involving a brief 1-min step-test (Woods et al. 2011b) and, second, at a simulated 4800 m over 3 h with a brief 5-min step-test (Boos et al. 2013). On neither occasion did we find a significant rise in BNP despite significant rises in PASP which we consider to be due to the brevity of the exercise stimulus. We have recently published the echocardiographic arm of this study (Boos et al. 2016) which reported that the right ventricular systolic pressure (RVSP) and pulmonary vascular resistance were higher in HH compared to NH. It would, therefore, be reasonable to expect that this would be associated with an elevation in NT-proBNP that we were unable to detect in HH. It is with great regret that we failed to get all the participants from the GHA through the HH exposure and we suspect that the lack of a rise in NT-proBNP under these conditions may simply reflect a type II error.

Although there appears, on the initial inspection of the data, to be some baseline variability in the parameters studied on statistical analysis, the only significant difference in resting values between environments was for PNORMET between NN and GHA. We suspect the higher resting PNORMET at GHA compared to NN relates to a degree of sympathoadrenal activation as a result of the cable car ascent to 3375 m on the morning of testing. As PMET and cortisol did not show any difference at baseline between NN and GHA and the fact there was still a significant increment in PNORMET with exercise at GHA, we suspect this was a minor confounder only. In support of this is the fact that all parameters at baseline in all environments were within the normal range expected for a healthy adult.

In summary, our exercise protocol produced significantly different results under conditions of NN versus the hypoxic environments. Exercise in the hypoxic environments successfully induced a measureable rise in several markers of physiological stress. From two-way ANOVA analysis, the changes seen between hypoxic environments were very similar with the only difference being for PNORMET. The previous comparisons between NH and HH have often focussed on respiratory or cardiovascular variables (Coppel et al. 2015), and while our data cannot be definitive, they have at least begun to evaluate equivalence of the environments from a perspective of biochemical and physiological stress.

Strengths of our study include the use of the same cohort of participants for each exposure with an adequate wash-out period between stimuli, with accurate reporting of barometric pressure to ensure homogeneity across environments. The major limitation was our inability, despite multiple attempts, to get all participants through the final HH exposure.

## Conclusion

To our knowledge, this is the first study to compare the effect of NN, NH, HH, and GHA in the same participants at rest and following exercise on a range of biochemical markers that reflect physiological stress. Responses under hypoxic conditions were different to NN, but the similarities between HH and NH suggest that they may be valid surrogates for GHA.

## Abbreviations

ANOVA: Analysis of variance
BNP: Brain natriuretic peptide
CV: Coefficient of variation
CLIA: Chemiluminescence immunoassay
ECG: Electrocardiograph
ECLIA: Electro-chemiluminescence immunoassay
FiO_2_: Fraction of inspired oxygen
GHA: Genuine high altitude
HA: High altitude
HH: Hypobaric hypoxia
hscTnT: Highly sensitive cardiac troponin T
LC–MSMS: Liquid chromatography mass spectrometry
NH: Normobaric hypoxia
NN: Normobaric normoxia
NT-proBNP: N-terminal pro-brain natriuretic peptide
PASP: Pulmonary artery systolic pressure
PiO_2_: Partial pressure of inspired oxygen
PMET: Plasma metanephrine
PNORMET: Plasma normetanephrine
RVSP: Right ventricular systolic pressure
SpO_2_: Peripheral oxygen saturation
QC: Quality control
Wmax: Maximal workload
